# A Conserved miR172-*TOE1* Module Coordinates Immunity and Flowering to Confer Verticillium Wilt Resistance in *Arabidopsis thaliana* and Cotton

**DOI:** 10.3390/plants15101567

**Published:** 2026-05-21

**Authors:** Ze Yu, Le Xu, Wambui Doris Njoki, Xiaoxiao Hu, Ran Wei, Ruonan Du, Cong Sheng, Muhammad Saqib Bilal, Isashova Umida, Hongwei Zhao

**Affiliations:** 1 State Key Laboratory of Agricultural and Forestry Biosecurity, College of Plant Protection, Nanjing Agricultural University, Nanjing 210095, China; 2019202024@njau.edu.cn (Z.Y.); 2020202033@stu.njau.edu.cn (L.X.); dorisnjokie@gmail.com (W.D.N.); 2023102056@stu.njau.edu.cn (X.H.); 2024102061@stu.njau.edu.cn (R.W.); ddruonan@163.com (R.D.); 2 Hunan Provincial Key Laboratory for Biology and Control of Plant Diseases and Insect Pests, College of Plant Protection, Hunan Agricultural University, Changsha 410128, China; csheng0531@163.com; 3 The Key Laboratory of Ecological Safety and Sustainable Development in Arid Lands, Xinjiang Institute of Ecology and Geography, Chinese Academy of Sciences, Urumqi 830011, China; msaqib@ms.xjb.ac.cn; 4 Department of Plant Protection, Andijan Agricultural and Agrotechnology Institute, Kuyganyor 170600, Uzbekistan; isashovaphd@mail.ru

**Keywords:** *TOE1*, miR172, VdsR-1, *A. thaliana*, cotton, Verticillium wilt resistance, delayed floral transition

## Abstract

Verticillium wilt, caused by *Verticillium dahliae*, is a devastating disease that severely threatens cotton production worldwide. The long-term survival of the pathogen in soil and the limited availability of resistant cultivars make effective control strategies challenging. Although the fungal cross-kingdom RNA VdsR-1 has been reported to delay floral transition and prolong vegetative growth, the underlying plant regulatory mechanisms remain largely unclear. Here, we show that the transcription factor *AtTOE1*, a target of ath-miR172b-3p, displays altered expression in response to changes in ath-miR172b-3p levels during *V. dahliae* inoculation, coinciding with coordinated changes in plant immune-related and developmental responses. Increased *AtTOE1* expression is correlated with enhanced disease resistance, reduced pathogen colonization, and delayed floral transition. Furthermore, our results indicate that the VdsR-1/*AtSPL13A* module is associated with modulation of *AtTOE1* expression via ath-miR172b-3p, suggesting the involvement of a cross-kingdom RNA-related regulatory framework linking plant immunity and development. Notably, this regulatory relationship is also observed in cotton, indicating evolutionary conservation across plant species. Together, our findings highlight *TOE1* as a potential integrator of defense and growth-related processes during pathogen challenge and provide insights that may inform strategies to improve resistance to *V. dahliae* in cotton and other crops.

## 1. Introduction

Cotton Verticillium wilt, caused by *V. dahliae*, is one of the most devastating diseases affecting cotton production worldwide. The pathogen invades the vascular system of cotton plants, causing leaf chlorosis, wilting, yield reduction, and inferior fiber quality, thereby severely constraining the production of stable, high-quality cotton [1]. Owing to the long-term soil persistence of the pathogen, the limited efficacy of chemical control, and the scarcity of highly resistant cultivars [2,3,4,5], effective management of Verticillium wilt remains a significant challenge in cotton production. Therefore, elucidating the pathogenesis and developing effective control strategies for Verticillium wilt is essential for ensuring stable and efficient cotton production, while also offering theoretical and technical foundations for cotton genetic improvement and sustainable agricultural development [6].

As a critical component of regulating plant-microbe interaction, miRNAs contribute to disease susceptibility or resistance by modulating gene expression, including pathogen recognition, activation of defense responses, and regulation of resistance gene expression [7]. In cotton, several miRNAs have been reported to participate in immune responses against Verticillium wilt. For example, the miR477-*CBP60A* module regulates the plant’s defense response by modulating salicylic acid (SA) signaling [8]. miR398b influences plant resistance to *V. dahliae* infection by regulating NBS-LRR and GhCSD genes, which are critical components of plant immune responses [9]. miR164 enhances cotton resistance to *V. dahliae* by regulating the *NAC100* transcription factor [10]. Cross-kingdom microRNAs miR166 and miR159 in cotton suppress *V. dahliae* infection by post-transcriptionally silencing the virulence genes *Clp-1* and *Hic-51* [11]. Moreover, it has been reported that *V. dahliae* regulates host development through small RNAs (sRNAs). Specifically, *V. dahliae* secretes VdsR-1 into host plants, where it associates with AGO1 to target the stem-loop junction of the miR157d precursor, promotes DCL1-mediated processing, increases the accumulation of miR157d, and represses *SPL13A/B* expression, thereby delaying the flowering transition [12]. This study reveals that *V. dahliae* infection significantly impacts plant growth and development and uncovers a previously underexplored regulatory role of miRNAs in mediating plant resistance to Verticillium wilt. These studies have contributed to clarifying the important role of miRNAs in cotton resistance to Verticillium wilt, partially addressing gaps in earlier research. Nevertheless, their scope has remained relatively narrow. Specifically, the underlying molecular mechanisms were not clearly elucidated, and the coordinated regulation between disease resistance and plant growth and development (agronomic traits) was not systematically examined.

The miR172b-*TOE1/2* module in *A. thaliana* suppresses the transcription of the immune receptor FLS2 via *TOE1/2*. As seedlings develop, miR172b levels increase while *TOE1/2* expression decreases, leading to the upregulation of FLS2 and enhanced immune responses [13]. miR172 suppresses *NtTOE3* expression through mRNA cleavage, whereas NtTOE3 directly binds to the promoters of the defense-related genes *KL1* and *MLP43*, activating their transcription and consequently enhancing tobacco resistance to TMV [14]. Moreover, TOE transcription factors are involved not only in plant immune regulation but also in governing plant growth and developmental processes [15,16]. During growth, development, and environmental adaptation, plants optimize resource allocation through precise cellular-level regulation, such as metabolic reprogramming and secondary metabolite biosynthesis, rather than relying solely on direct competition for energy [17,18]. A pronounced antagonistic interaction exists between jasmonic acid (JA) and gibberellic acid (GA): JA enhances defense responses by inhibiting cell elongation, whereas GA promotes growth by stimulating cell division and expansion [19,20]. The antagonistic interaction between JA and GA plays a pivotal role in balancing plant growth and defense. JA suppresses the biological activity of GA by activating DELLA proteins, which are negative regulators of the GA signaling pathway, thereby restraining growth while enhancing defense responses [21]. These findings suggest that *TOE1* plays a critical role in coordinating plant immune responses with growth and developmental processes.

Despite extensive studies on the miR172-*TOE1* module in developmental regulation, its potential role in coordinating plant immunity and growth during pathogen infection remains largely unclear. Therefore, this study aimed to investigate whether the miR172-*TOE1* regulatory pathway participates in responses to *V. dahliae* inoculation and how this module may integrate defense signaling with developmental processes. We focused on characterizing the regulatory relationship between ath-miR172b-3p and *AtTOE1* in *A. thaliana* and extended the analysis to its cotton homolog *GhTOE1* to evaluate potential conservation across species. In addition, we examined the possible involvement of the fungal cross-kingdom small RNA VdsR-1 and its interaction with the *SPL13A*/miR172 signaling cascade. To address these questions, we combined gene expression analyses, genetic manipulation, and pathogen inoculation assays to explore the regulatory framework linking plant immunity with growth regulation during *V. dahliae* inoculation.

## 2. Results

### 2.1. V. dahliae Induces AtTOE1 Expression via ath-miR172b-3p

**Figure 1:**
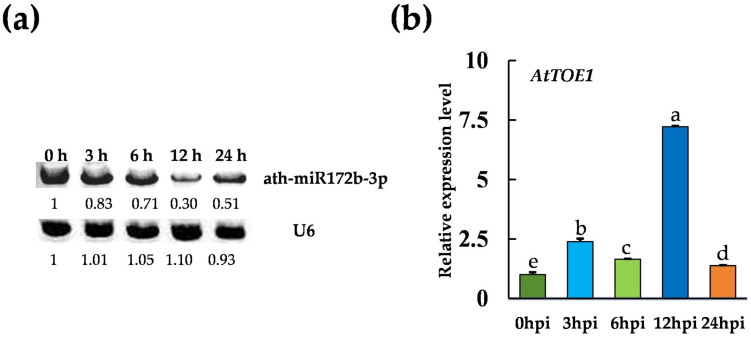
*V. dahliae* affects the expression of ath-miR172b-3p and *AtTOE1.* (**a**) Expression levels of ath-miR172b-3p upon *V. dahliae* inoculation. The expression levels were validated by RNA gel blot. The membrane was hybridized with biotin-labeled probes specific to ath-miR172b-3p. Detection of *A. thaliana* U6 (at-U6) small nuclear RNAs served as loading controls. (**b**) Relative expression levels of *AtTOE1* in *A. thaliana* after *V. dahliae* inoculation. Data are presented as means ± SD. Letters indicate significant differences (*p* < 0.05) determined by one-way analysis of variance (ANOVA) with Tukey’s test.

To explore the role of miRNAs in plant resistance to Verticillium wilt, small RNA sequencing analysis identified ath-miR172b-3p as a differentially expressed miRNA. The expression of ath-miR172b-3p was subsequently examined before and after *V. dahliae* inoculation, revealing that its abundance was significantly altered upon inoculation. Specifically, northern blotting showed that the expression level of ath-miR172b-3p progressively decreased during inoculation, dropping from a normalized value of 1.0 at 0 h post-inoculation to 0.3 at 12 hpi across the 0, 3, 6, 12, and 24 hpi time points (Figure 1a).

The putative target gene of ath-miR172b-3p, *AtTOE1*, was predicted using the psRNATarget tool. To verify this prediction, the 21 bp wild-type target sequence (wt) of *AtTOE1* and its mutant version (mu) were cloned into the 5′ end of the GFP reporter gene using an *Agrobacterium*-mediated transient expression system, generating the expression vectors 35S::*AtTOE1*wt-GFP and 35S::*AtTOE1*mu-GFP (Supplementary Figure S1a). These vectors were subsequently co-transformed into tobacco leaves together with either 35S::ath-miR172b-3p or the control vector 35S::ath-miR391. Fluorescence microscopy revealed that ath-miR172b-3p significantly suppressed *AtTOE1* expression compared with the synonymous substitution mutants, demonstrating the specificity of its regulatory activity (Supplementary Figure S1b). Increased *Agrobacterium* concentrations (0, 0.2, and 0.4 OD) were associated with a progressive reduction in fluorescence intensity, supporting a dose-dependent effect of miR172-mediated gene silencing. Western blot analysis mirrored the results observed in the fluorescence assays, showing a marked reduction in the protein levels of *AtTOE1* upon co-expression with ath-miR172b-3p (Supplementary Figure S1c). In contrast, no significant changes were detected upon co-expression of ath-miR172b-3p with the synonymous mutant constructs. RT-qPCR analysis revealed that *AtTOE1* expression was significantly reduced in the ath-miR172b-3p overexpression lines compared with Col-0 (Supplementary Figure S1d). Furthermore, analysis of *AtTOE1* expression after inoculation showed that *V. dahliae* significantly induced its transcription (Figure 1b). Overall, these findings suggest that *AtTOE1* expression is upregulated downstream of ath-miR172b-3p during *V. dahliae* inoculation.

### 2.2. AtTOE1 and GhTOE1 Enhance Resistance to V. dahliae in A. thaliana and Cotton

Most known functions of *AtTOE1* are associated with plant growth [22,23,24]. However, despite being a target of ath-miR172b-3p, its role in Verticillium wilt resistance remains largely unexplored. Notably, we also investigated *GhTOE1*, the cotton homolog of *AtTOE1*, to assess whether *TOE1* function is conserved across species. To investigate whether *GhTOE1* is involved in regulating resistance to *V. dahliae* in cotton, we analyzed its expression pattern following *V. dahliae* inoculation. RT-qPCR analysis showed that *V. dahliae* inoculation significantly induced *GhTOE1* expression at all examined time points (Supplementary Figure S2).

**Figure 2:**
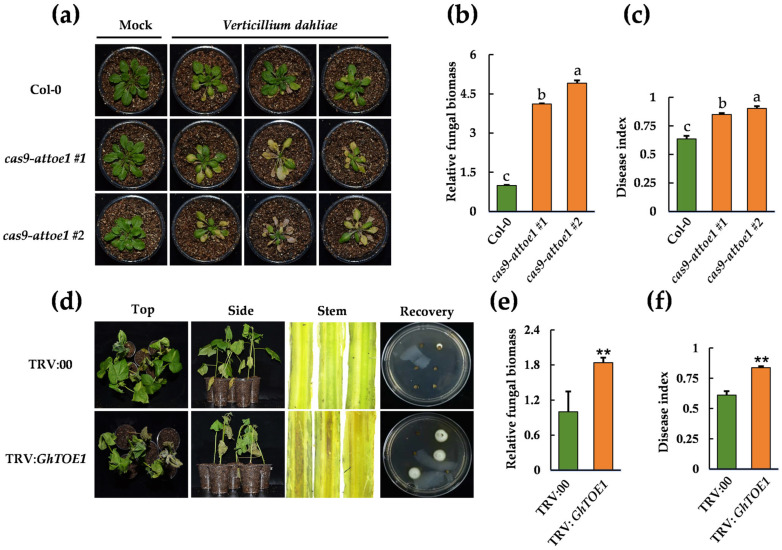
*AtTOE1* and *GhTOE1* are associated with *A. thaliana* and cotton resistance to *V. dahliae* inoculation, respectively. (**a**) Pathogenicity assays of Col-0 and *cas9-attoe1* mutants, with symptoms observed at 14 dpi. (**b**) Quantification of *V. dahliae* biomass in roots of Col-0 and *cas9-attoe1* mutants at 14 dpi. Data are presented as means ± SD. Letters indicate significant differences (*p* < 0.05) determined by one-way analysis of variance (ANOVA) with Tukey’s test. (**c**) The disease index of Col-0 and *cas9-attoe1* mutants at 14 dpi. Data are presented as means ± SD. Letters indicate significant differences (*p* < 0.05) determined by one-way analysis of variance (ANOVA) with Tukey’s test. (**d**) Disease symptoms in TRV:00 and TRV: *GhTOE1* plants at 14 dpi. Fungal recovery experiments and observation of necrotic vascular bundles in dissected stems. (**e**) Quantification of *V. dahliae* biomass in the stems of TRV:00 and TRV: *GhTOE1* plants at 14 dpi. Data are mean ± SD, and asterisks represent a significant difference with Student’s *t*-test (**, *p* < 0.01). (**f**) The disease index of TRV:00 and TRV: *GhTOE1* plants at 14 dpi. Data are mean ± SD, and asterisks represent a significant difference with Student’s *t*-test (**, *p* < 0.01).

To explore the biological function of *AtTOE1* in plant responses to *V. dahliae* inoculation, we constructed *AtTOE1* mutants, *cas9-attoe1*, in *A. thaliana* (Supplementary Figure S3). Compared to the wild type, the *cas9-attoe1* mutants exhibited increased disease symptoms (Figure 2a), with higher pathogen biomass (Figure 2b) and higher disease index (Figure 2c). To investigate the biological function of *GhTOE1*, a TRV-based virus-induced gene silencing (VIGS) cotton line targeting *GhTOE1* was constructed (Supplementary Figure S4). Similarly, when inoculated with *V. dahliae*, TRV: *GhTOE1* showed higher susceptibility (Figure 2d), with larger vascular bundle necrosis areas and greater amounts of the pathogen recovered from stem sections, compared to the TRV:00 control. Additionally, stems of TRV: *GhTOE1* plants contained significantly more fungal biomass compared with TRV:00 controls. (Figure 2e), and the disease index was also elevated (Figure 2f). These phenotypic differences suggest that *GhTOE1* also plays a positive regulatory role in cotton resistance to *V. dahliae* inoculation, and silencing it leads to decreased plant defense. This result is consistent with the induced expression of *AtTOE1* and *GhTOE1* by pathogens in *A. thaliana* and cotton (Figure 1b, Supplementary Figure S2), indicating that these genes participate in plant immune responses to vascular wilt pathogens.

### 2.3. AtSPL13A Directly Binds to the Promoter of ath-miR172b-3p

**Figure 3:**
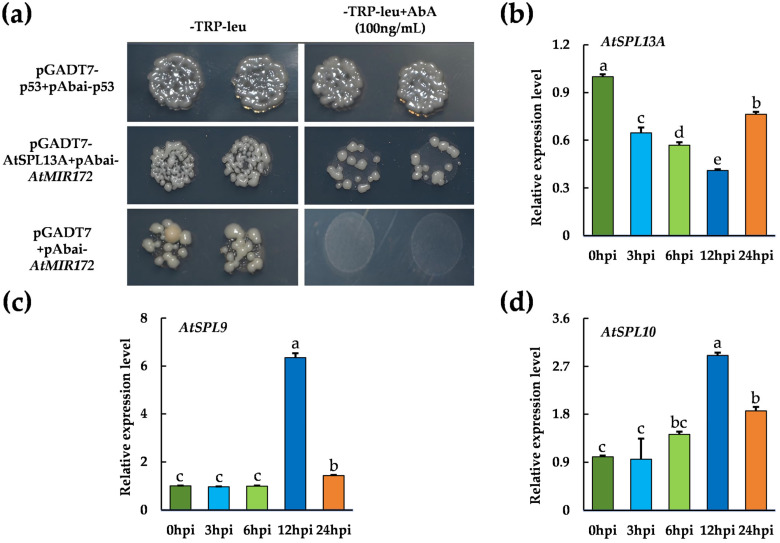
*AtSPL13A* regulates the *AtMIR172* promoter. (**a**) Y1H assay showing the interaction between *AtSPL13A* and *AtMIR172* promoter with controls: pGADT7-*AtSPL13A* and pABAi-*AtMIR172* (experimental), empty pGADT7 and pABAi-*AtMIR172* (negative control), pGADT7-p53 and pABAi-p53 (positive control). (**b**) Relative expression levels of *AtSPL13A* after inoculation with *V. dahliae.* (**c**) Relative expression levels of *AtSPL9* after *V. dahliae* inoculation. (**d**) Relative expression levels of *AtSPL10* after *V. dahliae* inoculation. Data are presented as means ± SD (**b**–**d**). Letters indicate significant differences (*p* < 0.05) determined by one-way analysis of variance (ANOVA) with Tukey’s test.

Previous research showed that SPLs regulate miR172 expression in a redundant manner [25,26,27]. Therefore, we were prompted to examine which SPL member plays a dominant role in regulating miR172 expression upon *V. dahliae* inoculation. To explore this regulatory relationship, the expression profiles of *AtSPL9*, *AtSPL10*, *AtSPL13A* and ath-miR172b-3p following *V. dahliae* inoculation were compared. The expression levels of *AtSPL9* and *AtSPL10* increased, peaking at 12 hpi, whereas *AtSPL13A* expression first declined and subsequently increased at 24 hpi post-inoculation, although it remained below the level observed at 0 hpi (Figure 3b,c). This *AtSPL13A* down-regulation correlated with a steady decrease in expression of ath-miR172b-3p very well (Figure 1a). Moreover, a Y1H assay was performed to examine the interaction between *AtSPL13A* and the *AtMIR172* promoter. Co-transformation of pGADT7-*AtSPL13A* and pABAi-*AtMIR172* enabled yeast growth on selective medium containing 100 ng/mL aureobasidin A (AbA), whereas the negative control, consisting of the empty pGADT7 vector and pABAi-*AtMIR172*, failed to grow under the same conditions. Y1H assay showed that AtSPL13A binds to the *AtMIR172* promoter (Figure 3a). Therefore, *AtSPL13A* may function as a transcription factor regulating ath-miR172b-3p expression upon *V. dahliae* inoculation.

### 2.4. Loss of VdsR-1 Correlates with Altered Host Immune-Related Responses During V. dahliae Infection

Based on previous findings, the fungal small RNA VdsR-1, secreted by *V. dahliae*, has been linked to altered expression of *AtSPL13A* through interference with the miR157d pathway, resulting in delayed flowering in *A. thaliana* [12]. In addition to its reported role in developmental regulation, our results indicate that the *AtSPL13A*/ath-miR172b-3p regulatory module is involved in changes in plant immune-related responses during interaction with *V. dahliae*. These observations prompted us to further explore the potential role of *AtSPL13A* at the interface between plant growth and immunity.

**Figure 4:**
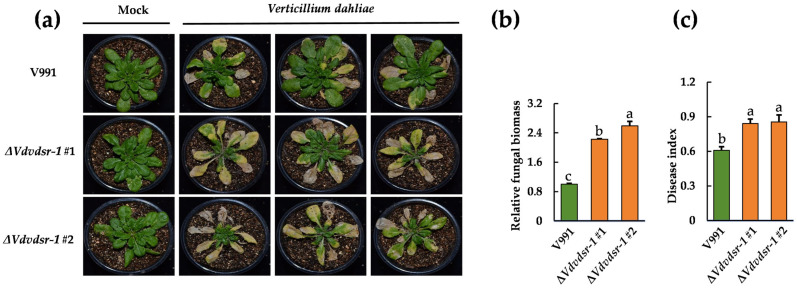
VdsR-1 modulates the pathogenicity of *V. dahliae*. (**a**) Phenotypes of *A. thaliana* infected with V991, Δ*Vdvdsr-1* #1 and Δ*Vdvdsr-1* #2 at 14 dpi. (**b**) Quantification of *V. dahliae* biomass in roots of *A. thaliana* infected with V991, Δ*Vdvdsr-1* #1 and Δ*Vdvdsr-1* #2 at 14 dpi. (**c**) The disease index of *A. thaliana* infected with V991, Δ*Vdvdsr-1* #1 and Δ*Vdvdsr-1* #2 at 14 dpi. Data are presented as means ± SD. Letters indicate significant differences (*p* < 0.05) determined by one-way analysis of variance (ANOVA) with Tukey’s test.

To this end, we generated a VdsR-1 deletion mutant of *V. dahliae* (Δ*Vdvdsr-1*; Supplementary Figure S5) and compared its inoculation outcomes with those of the wild-type strain V991. *A. thaliana* plants infected with the Δ*Vdvdsr-1* mutants developed more severe disease symptoms than those infected with V991 (Figure 4a). Consistently, quantification of fungal biomass revealed higher levels of *V. dahliae* colonization in roots of plants infected with the Δ*Vdvdsr-1* mutants (Figure 4b), and the disease index was significantly increased relative to V991-infected controls (Figure 4c). Together, these results indicate that the loss of VdsR-1 correlates with enhanced disease severity and increased host susceptibility during *V. dahliae* inoculation.

### 2.5. VdsR-1 and SPL13A Are Associated with Altered miR172 Expression During V. dahliae Infection

**Figure 5:**
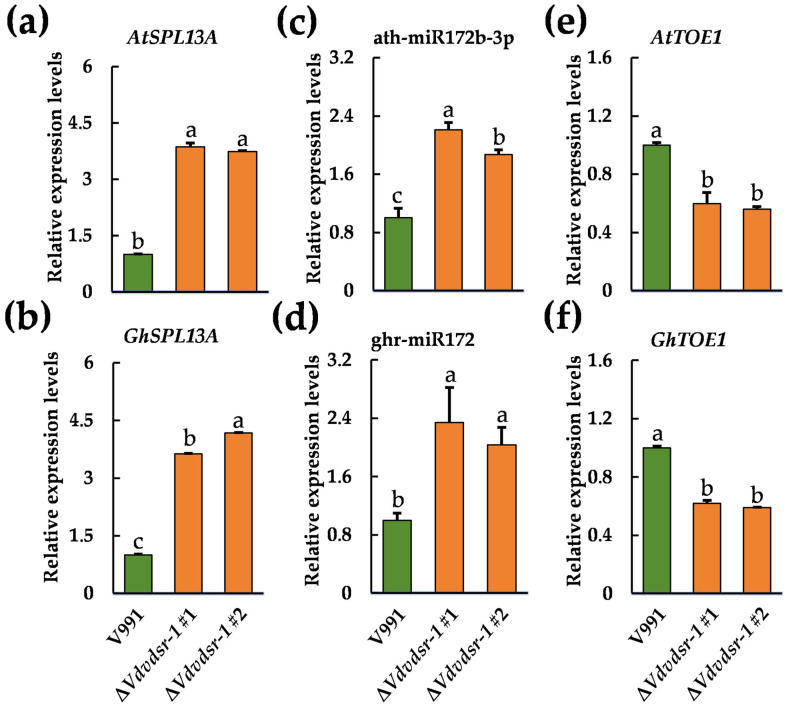
VdsR-1 regulates miR172 and *TOE1* expression. (**a**–**f**) Relative expression levels of *AtSPL13A*, *GhSPL13A*, ath-miR172-3p, ghr-miR172, *AtTOE1* and *GhTOE1* in the *A. thaliana* and cotton infected with V991, Δ*Vdvdsr-1* #1 and Δ*Vdvdsr-1* #2 at 24 hpi. Data are presented as means ± SD. Letters indicate significant differences (*p* < 0.05) determined by one-way analysis of variance (ANOVA) with Tukey’s test.

To explore the regulatory effects associated with VdsR-1 during *V. dahliae* inoculation, we analyzed the expression levels of *AtSPL13A* and ath-miR172b-3p in *A. thaliana*, as well as *GhSPL13A* and ghr-miR172 in cotton plants infected with the V991, ΔVdvdsr-1 #1, and ΔVdvdsr-1 #2 strains. We found that the transcript levels of both *AtSPL13A* and *GhSPL13A* were significantly higher in plants infected with the Δ*Vdvdsr-1* mutants than in those infected with the wild-type strain (Figure 5a,b). Likewise, transcript quantities of ath-miR172b-3p and ghr-miR172 increased significantly in plants inoculated with the Δ*Vdvdsr-1* mutants relative to the wild-type control plant (Figure 5c,d). These expression patterns indicate that the absence of VdsR-1 is associated with altered regulation of the *SPL13A*-miR172 module during inoculation.

We further examined the expression of *AtTOE1* and *GhTOE1* in the same plant materials. Consistent with changes observed in upstream regulatory components, both *AtTOE1* and *GhTOE1* transcript levels were significantly lower in plants infected with the ΔVdvdsr-1 strains (Figure 5e,f). Together, these results support a model in which VdsR-1 influences the *SPL13A*/miR172/*TOE1* regulatory module at the transcriptional level, thereby affecting host developmental and immune-related responses during *V. dahliae* inoculation. Notably, similar regulatory trends were observed in both *A. thaliana* and cotton, suggesting functional conservation of this module across species. Rather than indicating identical regulatory circuitry or co-evolutionary adaptation, these findings point to a conserved regulatory outcome linking developmental modulation with plant responses to *V. dahliae* inoculation.

### 2.6. TOE1 Is Involved in Transcriptional Changes in Growth-And Development-Related Genes During Infection

**Figure 6:**
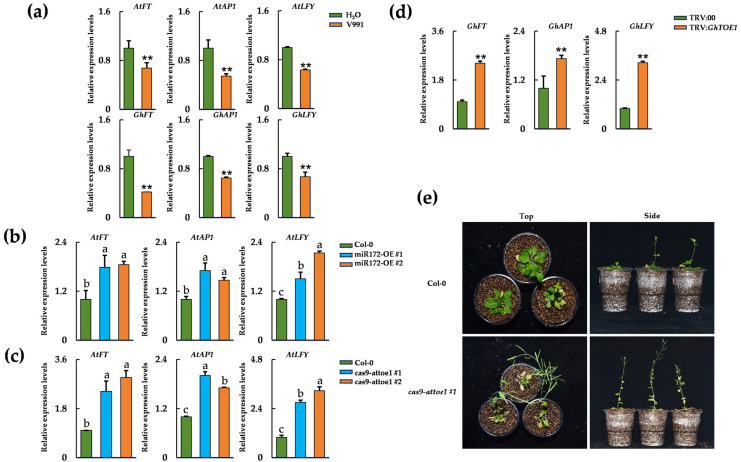
*TOE1* coordinates disease-associated responses and growth-related regulation in *A. thaliana* and cotton. (**a**) Relative expression levels of *FT*, *AP1*, and *LFY* in *A. thaliana* and cotton seedlings 24 h after treatment with H_2_O and V991. Data are mean ± SD and asterisks represent significant difference with Student’s *t*-test (**, *p* < 0.01). (**b**) Relative expression levels of *AtFT*, *AtAP1*, and *AtLFY* in Col-0 and miR172-OE lines (#1 and #2) at 24 hpi. Data are presented as means ± SD. Letters indicate significant differences (*p* < 0.05) determined by one-way analysis of variance (ANOVA) with Tukey’s test. (**c**) Relative expression levels of *AtFT*, *AtAP1*, and *AtLFY* in Col-0 and *cas9-attoe1* mutants at 24 hpi. Data are presented as means ± SD. Letters indicate significant differences (*p* < 0.05) determined by one-way analysis of variance (ANOVA) with Tukey’s test. (**d**) Relative expression levels of *GhFT*, *GhAP1*, and *GhLFY* in TRV:00 and TRV: *GhTOE1* plants at 24 hpi. Data are mean ± SD, and asterisks represent a significant difference with Student’s *t*-test (**, *p* < 0.01). (**e**) The flowering phenotypes of Col-0 and *cas9-attoe1* mutants after inoculation with V991.

Previous studies have shown that *AtTOE1* can directly regulate the expression of developmental genes such as *AtFT*, *AtLFY*, and *AtAP1* [28,29,30]. Based on this regulatory relationship, we analyzed whether the expression of these genes was altered during *V. dahliae* inoculation and whether such changes were associated with *AtTOE1* activity. RT-qPCR analysis revealed that at 24 hpi, the transcript levels of *AtFT*, *AtLFY*, and *AtAP1* were significantly reduced in *A. thaliana* compared with uninfected controls. A similar expression pattern was observed in cotton, where *GhFT*, *GhLFY*, and *GhAP1* transcript levels also decreased following *V. dahliae* inoculation (Figure 6a). These observations are consistent with increased *TOE1* accumulation coinciding with reduced expression of flowering-related genes during inoculation.

To further assess the role of *TOE1*, we analyzed gene expression in cas9-attoe1 mutants and TRV: *GhTOE1*-treated cotton plants. In both systems, the expression of *FT*, *LFY*, and *AP1* was significantly higher than that in wild-type or TRV:00-treated plants (Figure 6c,d). In addition, ath-miR172b-3p-overexpressing *A. thaliana* lines displayed elevated expression of these genes relative to the wild type (Figure 6b). These results support a regulatory role for the miR172-*TOE1* module in modulating the expression of flowering-related genes in both *A. thaliana* and cotton. Consistent with these transcriptional changes, *attoe1* mutants exhibited accelerated flowering following inoculation with the V991 strain (Figure 6e). Collectively, these findings support a model in which *V. dahliae* inoculation suppresses developmental programs controlled by *FT*, *LFY*, and *AP1*, leading to delayed flowering through the miR172-*TOE1* regulatory pathway.

## 3. Discussion

miRNAs play pivotal roles in plant disease resistance by regulating genes involved in pathogen perception, defense activation, and immune signaling pathways [7,31,32,33]. Consistent with previous studies demonstrating miR172 involvement in plant immunity [14,34,35], we observed that *V. dahliae* inoculation reduces ath-miR172b-3p abundance in *A. thaliana*, accompanied by increased expression of its target gene *AtTOE1*. This supports a regulatory model in which the ath-miR172b-3p/*AtTOE1* module participates in host responses to *V. dahliae* inoculation. Notably, a similar regulatory trend was observed in cotton, suggesting a partial functional conservation of this module across species. The miR172/*TOE1* pathway has also been implicated in controlling the vegetative-to-floral transition [23], and our findings are consistent with the idea that extended vegetative growth may influence susceptibility to *V. dahliae*, highlighting a potential growth-defense trade-off. Furthermore, genetic and transcriptional evidence positions the *SPL13A*/miR172/*TOE1* module downstream of the VdsR-1/miR157d pathway, in agreement with prior observations in *A. thaliana* and other plant-pathogen systems [12,25,26,27]. Collectively, these results suggest that, in addition to activating canonical defense responses, plants may reprogram developmental and resource allocation pathways as part of an integrated strategy to cope with *V. dahliae* inoculation, reinforcing the notion that immune responses and developmental regulation are tightly interconnected.

Previous studies have implicated *TOE1* in the regulation of plant immune responses [13,35]. Consistent with these reports, our findings suggest that *TOE1* contributes to resistance against Verticillium wilt. However, rather than defining a single linear defense mechanism, our results support a broader conceptual framework in which modulation of the miR172-*TOE1* module links developmental regulation with disease responses. Previous studies have demonstrated that *TOE1* represses flowering by regulating key integrator genes such as *FT*, *LFY*, and *AP1* [28,29,30], thereby controlling the vegetative-to-reproductive transition. In agreement with this developmental role, we observed that alteration of the miR172-*TOE1* module was associated with delayed flowering, suggesting that developmental timing may influence disease susceptibility. Such developmental reprogramming has been reported in other plant-pathogen interactions; for example, the fungal small RNA VdsR-1 enhances miR157d accumulation while repressing *SPL13A/B* expression during *A. thaliana* infection, leading to dwarfism and delayed flowering [12]. Furthermore, several SPL family members, including *SPL9*, *SPL10*, and *SPL13*, have been shown to promote miR172 expression [25,26,27], indicating a regulatory network that integrates developmental phase transitions with stress responses. Collectively, these observations support the idea that plant growth and developmental programs, in addition to direct immune signaling, may shape resistance outcomes against *V. dahliae* by influencing physiological states that are less favorable for pathogen progression.

VdsR-1 is a fungal-derived small RNA that has been shown to modulate flowering time in *A. thaliana* by interfering with the miR157d/*SPL13A* pathway [12]. Our results indicate that key components of the VdsR-1/*SPL13A* module exhibit similar regulatory trends in cotton, suggesting partial functional conservation rather than identical network architecture (Figure 6). Importantly, this conservation should be interpreted at the level of regulatory trends and biological outcomes, rather than as evidence for a fully conserved molecular network across species. Given the substantial evolutionary divergence between *Arabidopsis* and cotton, differences in upstream regulatory factors and gene regulatory network topology are expected, even when similar phenotypic outputs are observed, as regulatory divergence and network rewiring have been widely documented in cotton and other plant lineages [36,37,38]. The consistent phenotypes observed across independent Δ*Vdvdsr-1* mutant lines provide a non-redundant genetic definition of the VdsR-1 loss-of-function phenotype, supporting a specific contribution of this small RNA to fungal pathogenicity during host infection.

Based on our genetic and transcriptional analyses, VdsR-1 appears to influence both plant developmental programs and immune-related responses. Rather than acting as a classical virulence effector, VdsR-1 may function as a modulator of host developmental timing, with downstream effects on the immune-developmental balance. Reduced *SPL13A* expression correlates with altered miR172 levels and consequent changes in downstream gene expression, including *TOE1*. These molecular adjustments are associated not only with direct resistance phenotypes but also with developmental alterations, such as delayed floral transition. Collectively, these findings support a conceptual framework in which pathogen-derived small RNAs can reprogram host developmental trajectories to indirectly modulate disease susceptibility, illustrating the tight integration of developmental and immune networks in plant-pathogen interactions.

Effective disease resistance strategies should be compatible with agronomic traits to enable practical application in crop improvement [39]. Our study proposes a regulatory framework that integrates direct defense responses with developmental modulation, highlighting the importance of coordination between growth and immunity. Although resistance phenotypes were primarily evaluated at the seedling and early vegetative stages, these findings emphasize the relevance of growth stage-dependent resistance mechanisms, which have been widely reported in plant-pathogen interactions [40,41,42,43]. Previous studies have shown that plants often exhibit distinct immune capacities during vegetative and reproductive phases, suggesting that developmental timing can influence disease outcomes [44]. In this context, modulation of developmental programs may represent an adaptive strategy to optimize resource allocation between growth and defense. The framework described here connects immune signaling with developmental regulation and may provide a conceptual basis for identifying breeding-relevant targets aimed at enhancing disease resistance without compromising crop performance. Despite these advances, several limitations should be acknowledged. Although this study provides evidence supporting the role of the miR172-*TOE1* regulatory module, functional validation through *GhTOE1* overexpression in cotton was not performed in the present work. Future studies involving stable or transient overexpression assays in cotton will be necessary to further confirm the biological function of *GhTOE1* in cotton defense responses. In addition, the long-term agronomic consequences of manipulating this pathway remain unclear, and potential trade-offs associated with delayed flowering or altered developmental timing will require further evaluation under field or near-field conditions to assess their practical feasibility.

## 4. Materials and Methods

### 4.1. Plant Materials and Growth Conditions

Wild-type *A. thaliana* (Col-0) and transgenic *A. thaliana* seeds were initially germinated in vermiculite. Seven days later, seedlings were transferred to a soil substrate consisting of nutrient soil and vermiculite mixed at a ratio of 1:2 and cultivated under controlled conditions with a 12 h light/12 h dark cycle at 22–24 °C. *Nicotiana benthamiana* seeds were also germinated in vermiculite and, after 7 days, transplanted into fresh vermiculite. The environmental conditions used for *N. benthamiana* growth were the same as those applied to *A. thaliana*. Seeds of wild-type cotton (TM-1) were immersed in sterile distilled water (SDW) and incubated in darkness at 28 °C for 3 days to promote germination. Subsequently, the seedlings were transferred to a soil mixture containing nutrient soil and vermiculite at a 1:1 ratio. Cotton plants were grown under the same conditions as *A. thaliana*.

### 4.2. Pathogen Cultivation, Plant Inoculation and Disease Assay

The cultivation of *V. dahliae* strain V991 was carried out as previously reported [45]. *V. dahliae* was cultured on potato dextrose agar (PDA) plates prepared with 200 g peeled potato, 20 g D-glucose (Sinopharm Chemical Reagent Co., Ltd., Shanghai, China; Cat. No. 63005518), and 15–20 g agar powder (YEASEN, Shanghai, China; Cat. No. 70101ES76) per 1000 mL distilled water. The medium was sterilized at 121 °C for 15–20 min. Plates were incubated at 28 °C in the dark for 2 weeks. Mycelial plugs were aseptically excised and transferred into liquid PDA medium (PDA plates without agar powder), followed by incubation at 28 °C with shaking at 200 rpm for 48 h to induce sporulation. The resulting culture was filtered through sterile filter paper to remove mycelial debris. The spore concentration was determined using a hemocytometer under a light microscope and adjusted to 1 × 10^7^ spores/mL with sterile distilled water. For inoculation, three-week-old *A. thaliana* seedlings were gently removed from the substrate, and their roots were submerged in the spore suspension for 10 min prior to transplantation into a soil mixture of nutrient soil and vermiculite (1:2, *v*/*v*). Cotton seedlings at the four-leaf stage were inoculated by immersing the roots in the spore suspension for 1 h and subsequently transferred to a soil mixture containing nutrient soil and vermiculite at a ratio of 1:1. In all inoculation assays, SDW was used as a mock treatment control for both *A. thaliana* and cotton. Each treatment included at least 20 plants. Disease severity was evaluated and recorded according to established criteria [46].

### 4.3. DNA and RNA Extraction, and Quantitative Analysis

Total RNA was isolated from *A. thaliana* seedlings using TRIzol reagent (Invitrogen, Carlsbad, CA, USA; Cat. No. 89880), whereas RNA from cotton tissues was extracted with the RNApure Fast Plant Kit (CWBio, Taizhou, China; Cat. No. CW0598S). Genomic DNA from *A. thaliana* was prepared using the cetyltrimethylammonium bromide (CTAB) method, while cotton genomic DNA was obtained with the FastClean Plant Genomic DNA Kit (CWBio, Taizhou, China; Cat. No. CW0571S).

The purity and concentration of all RNA and DNA samples were evaluated using a NanoDrop 1000 spectrophotometer (Thermo Fisher Scientific, Waltham, MA, USA) to confirm their suitability for subsequent analyses. First-strand complementary DNA (cDNA) was synthesized from total RNA using the PrimeScript RT Reagent Kit (TaKaRa, Kusatsu, Japan; Cat. No. RR047A). Quantitative real-time PCR (RT-qPCR) and genomic DNA-based qPCR assays were conducted with 2× AceQ^®^ qPCR SYBR^®^ Green Master Mix (Vazyme, Nanjing, China; Cat. No. Q711-02). The relative expression levels were calculated using the 2^−ΔΔCt^ method. The primers used for quantitative amplification are provided in Supplementary Table S1.

### 4.4. Transient Expression Assay in N. benthamiana

To assess GFP reporter gene expression, 21 bp target sequences of *AtTOE1*, along with their corresponding mutated versions, were fused to the 5′ end of the GFP coding sequence using conventional cloning approaches. These fusion constructs were subsequently inserted into the pBIN-3HA vector via restriction enzyme-mediated ligation. Precursor sequences of ath-miR172-3p and ghr-miR172 were cloned into the pNC-Cam3304-35S vector employing the Nimble Cloning Reagent kit (NC Biotech, Haikou, China; Cat. No. NC001).

The resulting recombinant vectors were transformed into *Agrobacterium tumefaciens* strain GV3101 (Daling Bio, Beijing, China; Cat. No. DLC305) and used for co-infiltration into *N. benthamiana* leaves. Fluorescence signals were imaged 48 h post-infiltration using an Axio Observer 3 microscope (Carl Zeiss, Oberkochen, Germany). For subsequent protein analysis, total proteins were extracted from the infiltrated leaves and subjected to Western blotting.

### 4.5. Northern Blot

Total RNA (25 µg) was mixed with an equal volume of 2× RNA loading buffer (Beyotime, Shanghai, China; Cat. No. R0215) and resolved on a 14% denaturing urea-polyacrylamide gel. Electrophoresis was performed at 70 V for 7 h. Separated RNA was transferred onto a Hybond-N+ membrane (Cytiva, Marlborough, MA, USA; Cat. No. RPN303B) using a wet transfer system. Membranes were hybridized with 5′-biotin-labeled probes at 37 °C for 24 h, with U6 as the internal control, followed by washing with 0.1% SDS in 20× SSC. Signals were detected using a chemiluminescent nucleic acid detection kit (Thermo Fisher Scientific, Waltham, MA, USA; Cat. No. 89880), and probe sequences are provided in Supplementary Table S1.

### 4.6. Virus-Induced Gene Silencing in Cotton

Virus-induced gene silencing (VIGS) assays were performed using the pTRV1 and pTRV2 vectors as previously described [46]. The coding sequence of *GhTOE1* was individually inserted into the pTRV2 vector. Total RNA was isolated from newly emerged leaves, and gene silencing efficiency was evaluated by RT-qPCR. Following confirmation of effective silencing, the silenced plants were subjected to *V. dahliae* inoculation assays.

### 4.7. Construction of Recombinant Vectors and Genetic Transformation of Plants

To generate a CRISPR/Cas9 construct targeting *AtTOE1*, single-guide RNAs (sgRNAs) were designed using an online design platform (http://skl.scau.edu.cn/, accessed on 19 October 2025). Primers specific to *AtTOE1* were used to amplify the corresponding sgRNA fragments from the pCBC-DT1T2 template, which were subsequently inserted into the pNC-HSE401 vector. The resulting recombinant plasmid, carrying hygromycin B resistance as a selection marker, was introduced into *A. thaliana* ecotype Col-0 via *Agrobacterium*-mediated transformation. To confirm mutations at the target loci, genomic DNA was isolated from T2-generation plants, and fragments of approximately 500–1000 bp encompassing the target sites were amplified by PCR. The amplified products were sequenced, and the resulting sequences were aligned with the wild-type reference genome to identify genome-editing events.

The 5′ and 3′ flanking regions (2000 bp each) of the *MIR-VdsR-1* sequence were inserted into the pGKO vector, after which the resulting construct was introduced into *A. tumefaciens* strain EHA105 (Daling Bio, Beijing, China; Cat. No. DLC308). A mixture of *A. tumefaciens* EHA105 and V991 spores was then co-cultured, and transformants were screened using hygromycin B selection and verified by qPCR.

### 4.8. Y1H Assay

A 2.0 kb fragment corresponding to the promoter region of *AtMIR172* was amplified and cloned into the pAbAi vector. The resulting construct was linearized and transformed into the Y1HGold (WEIDI, Shanghai, China; Cat. No. YC1001S) yeast strain to generate the bait strain pAbAi-*AtMIR172*. The minimum inhibitory concentration (MIC) of aureobasidin A (YEASEN, Shanghai, China; Cat. No. 60231ES03) for each bait strain was subsequently determined. The coding sequence of *AtSPL13A* was cloned into the pGADT7 vector to generate the prey construct. Y1H assays were then performed according to the manufacturer’s instructions. The primer sequences used for the Y1H assays are listed in Supplementary Table S1.

### 4.9. Western Blot

Total proteins were extracted using 5× SDS-PAGE sample loading buffer composed of 250 mM Tris-HCl (solarbio, Beijing, China; Cat. No. T8230), 10% SDS (biosharp, Beijing, China; Cat. No. BS088-500g), 0.5% bromophenol blue (YEASEN, Shanghai, China; Cat. No. 60503ES08), 50% glycerol (GHTECH, Guangzhou, China; Cat. No. 1.17062.020), and 5% β-mercaptoethanol (YEASEN, Shanghai, China; Cat. No. 52644ES03), pH 6.8. Protein signals were detected with the SuperPico ECL Chemiluminescence Kit (Vazyme, Nanjing, China; Cat. No. E422-01/02) using an anti-GFP antibody (Abmart, Shanghai, China; Cat. No. ab13970). Ponceau S staining of membranes was applied as a loading control to assess total protein levels.

### 4.10. Statistical Analysis

Experimental data were analyzed using GraphPad Prism (10.1.2). All experiments were conducted with at least three independent biological replicates, and data are expressed as mean ± standard deviation (SD). Statistical significance among multiple groups was determined by one-way analysis of variance (ANOVA) followed by Tukey’s test (*p* < 0.05). Significant differences were analyzed using Student’s *t*-tests for pairwise comparisons. Significance levels are denoted as follows: **, *p* < 0.01; ns, not significant.

Quantification of grayscale intensity was carried out with ImageJ software (v1.52i). Equal-sized regions of interest (ROIs) were selected for all samples under identical exposure conditions. Background subtraction was performed prior to the calculation of integrated density, and the obtained values were normalized to the loading control.

## 5. Conclusions

In conclusion, this study highlights *TOE1* as an important regulatory component linking plant immune responses and developmental processes during *V. dahliae* inoculation. Our results indicate that suppression of miR172 following pathogen challenge is accompanied by increased *TOE1* expression, which is associated with enhanced disease resistance, reduced fungal colonization, and delayed floral transition. These observations suggest that *TOE1* is not only involved in developmental regulation but may also participate in the modulation of host defense-related responses. In addition, our findings indicate that the fungal cross-kingdom RNA VdsR-1, acting through the *SPL13A* pathway, is associated with changes in *TOE1* accumulation via regulation of miR172 expression, linking pathogen-derived RNA signals to host transcriptional responses. Together, these results support a model in which *TOE1*-associated regulatory networks contribute to the coordination of growth-defense trade-offs during *V. dahliae* inoculation. This work provides insights into cross-kingdom RNA-related regulatory interactions and may inform future strategies for improving resistance to Verticillium wilt in cotton and other crops.

## Data Availability

The original contributions presented in the study are included in the article and Supplementary Materials; further inquiries can be directed to the corresponding author.

## Supplementary Materials

The following supporting information can be downloaded at https://www.mdpi.com/article/10.3390/plants15101567/s1: Figure S1: ath-miR172b-3p suppresses the expression of *AtTOE1*. (**a**) Schematics of *AtTOE1*wt (containing *AtTOE1* target sites)-green fluorescent protein (GFP) and *AtTOE1*mu (containing mutated *AtTOE1* target sites)-GFP fusion proteins, and alignment of the sequences of *AtTOE1*wt and *AtTOE1*mu with ath-miR172b-3p. (**b**) Fluorescence of the reporter gene in a *Nicotiana benthamiana* transient expression assay showing that ath-miR172b-3p silenced *AtTOE1*, but not its mutated target sites, *AtTOE1*mu. According to the psRNATarget prediction, ath-miR391 does not target *AtTOE1* and was used as a negative control. Scale bars = 200 μm. (**c**) Western blot results showing that ath-miR172b-3p silenced *AtTOE1*, but not its mutated target sites, *AtTOE1*mu. According to the psRNATarget prediction, ath-miR391 does not target *AtTOE1* and was used as a negative control. (**d**) Expression levels of *AtTOE1* in Col-0 and miR172-OE lines. Data are presented as means ± SD. Letters indicate significant differences (*p* < 0.05) determined by one-way analysis of variance (ANOVA) with Tukey’s test. Figure S2: *Verticillium dahliae* induces the expression of *GhTOE1.* Relative expression levels of *GhTOE1* in cotton after *V. dahliae* inoculation. Data are presented as means ± SD. Letters indicate significant differences (*p* < 0.05) determined by one-way analysis of variance (ANOVA) with Tukey’s test. Figure S3: Validation of *cas9-attoe1* mutant lines. (**a**) Transgenic plants were selected by spraying with glufosinate-ammonium. (**b**) Sequence validation of CRISPR/Cas9-generated *attoe1* mutant lines. Figure S4: Validation of VIGS-derived cotton silencing lines. (**a**) Cotton leaves were infiltrated with TRV: *GhCLA1*, TRV:00 (empty vector control), or TRV: *GhTOE1*. Plants treated with TRV: *GhCLA1* served as a positive control and exhibited the expected leaf bleaching phenotype. Phenotypes were assessed at 14 days post-inoculation (dpi). (**b**) The relative expression levels of *GhTOE1* in TRV:00 and TRV: *GhTOE1* plants were determined by RT-qPCR. Data are mean ± SD and asterisks represent significant difference with Student’s *t*-test (**, *p* < 0.01). Figure S5: Validation of the VdsR-1 knockout mutant. (**a**) Positive transformants were confirmed by PCR detection of the hygromycin resistance gene. (**b**) Relative expression levels of VdsR-1 in V991, Δ*Vdvdsr-1 #1*, and Δ*Vdvdsr-1 #2* strains. Data are presented as means ± SD. Letters indicate significant differences (*p* < 0.05) determined by one-way analysis of variance (ANOVA) with Tukey’s test. Table S1. Primers used in the study.
