# Supplementary material for: A Conserved miR172-TOE1 Module Coordinates Immunity and Flowering to Confer Verticillium Wilt Resistance in Arabidopsis thaliana and Cotton
Source: Plants (Basel). 2026 May 21;15(10):1567. doi: 10.3390/plants15101567 (PMC13210697; doi:10.3390/plants15101567)
Supplement: Supplementary file 1 [file plants-15-01567-s001.zip › Supplementary-Figure S1-5.pdf]

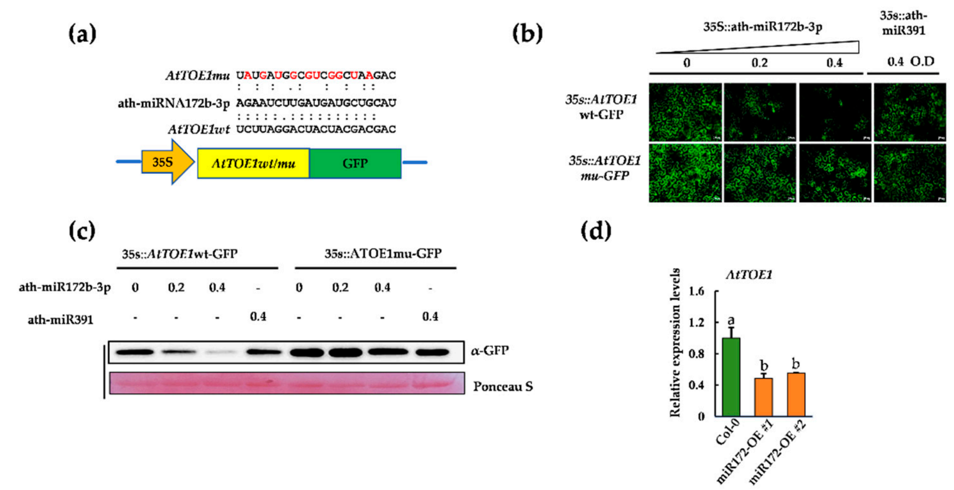

Figure S1: ath-miR172b-3p suppresses the expression of *AtTOE1*. (**a**) Schematics of *AtTOE1*wt (containing *AtTOE1* target sites)-green fluorescent protein (GFP) and *AtTOE1*mu (containing mutated *AtTOE1* target sites)-GFP fusion proteins, and alignment of the sequences of *AtTOE1*wt and *AtTOE1*mu with ath-miR172b-3p. (**b**) Fluorescence of the reporter gene in a *Nicotiana benthamiana* transient expression assay showing that ath-miR172b-3p silenced *AtTOE1*, but not its mutated target sites, *AtTOE1*mu. According to the psRNATarget prediction, ath-miR391 does not target *AtTOE1* and was used as a negative control. Scale bars = 200 μm. (**c**) Western blot results showing that ath-miR172b-3p silenced *AtTOE1*, but not its mutated target sites, *AtTOE1*mu. According to the psRNATarget prediction, ath-miR391 does not target *AtTOE1* and was used as a negative control. (**d**) Expression levels of *AtTOE1* in Col-0 and miR172-OE lines. Data are presented as means ± SD. Letters indicate significant differences ($p < 0.05$) determined by one-way analysis of variance (ANOVA) with Tukey's test.

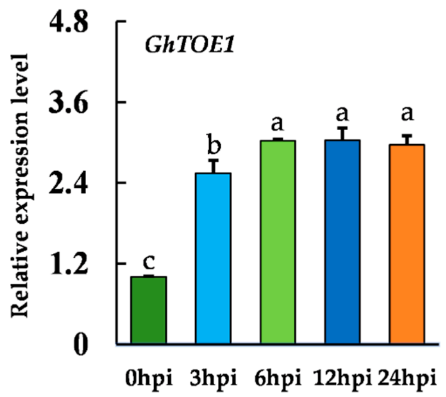

Figure S2: *Verticillium dahliae* induces the expression of *GhTOE1*. Relative expression levels of *GhTOE1* in cotton after *V. dahliae* inoculation. Data are presented as means ± SD. Letters indicate significant differences ($p < 0.05$) determined by one-way analysis of variance (ANOVA) with Tukey's test.

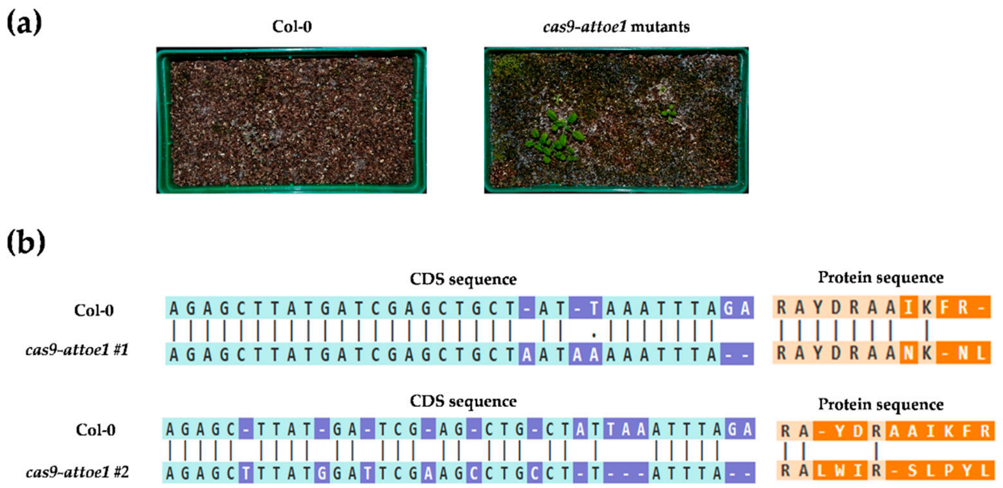

Figure S3: Validation of *cas9-attoe1* mutant lines. (**a**) Transgenic plants were selected by spraying with glufosinate-ammonium. (**b**) Sequence validation of CRISPR/Cas9-generated *attoe1* mutant lines.

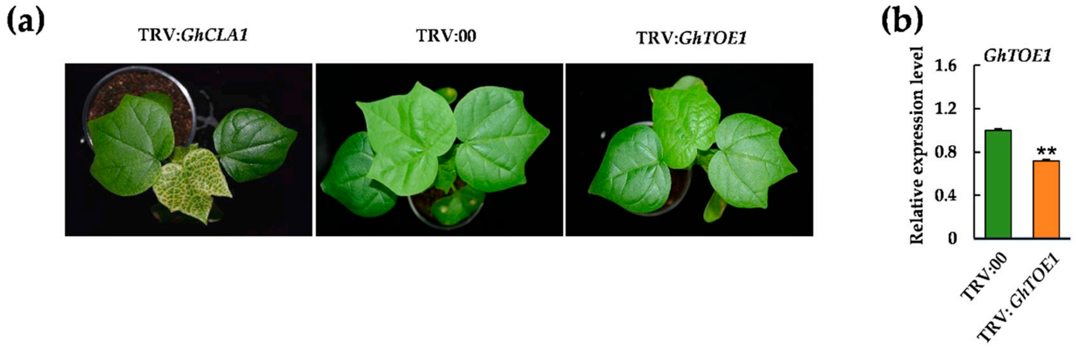

Figure S4: Validation of VIGS-derived cotton silencing lines. (**a**) Cotton leaves were infiltrated with TRV: *GhCLA1*, TRV:00 (empty vector control), or TRV: *GhTOE1*. Plants treated with TRV: *GhCLA1* served as a positive control and exhibited the expected leaf bleaching phenotype. Phenotypes were assessed at 14 days post-inoculation (dpi). (**b**) The relative expression levels of *GhTOE1* in TRV:00 and TRV: *GhTOE1* plants were determined by RT-qPCR. Data are mean ± SD and asterisks represent significant difference with Student's t-test (**, $p < 0.01$).

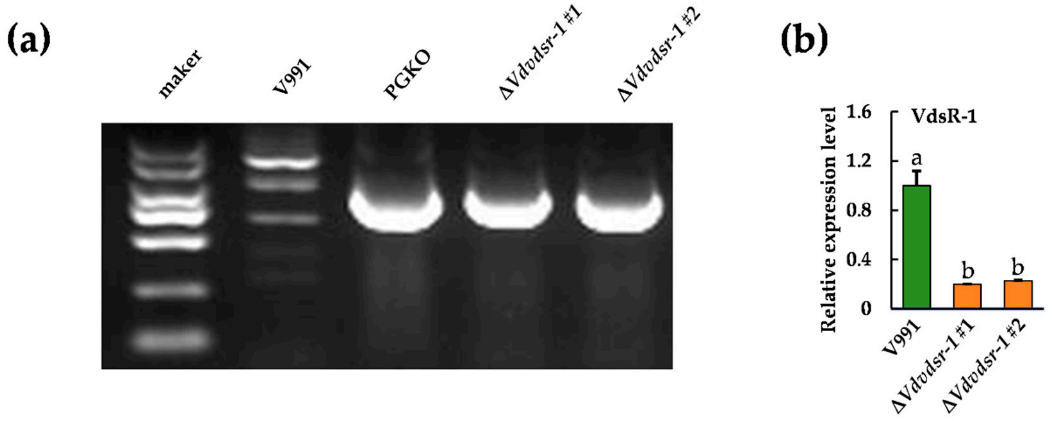

Figure S5: Validation of the VdsR-1 knockout mutant**.** (**a**) Positive transformants were confirmed by PCR detection of the hygromycin resistance gene. (**b**) Relative expression levels of VdsR-1 in V991, Δ*Vdvdsr-1* #1, and Δ*Vdvdsr-1* #2 strains. Data are presented as means ± SD. Letters indicate significant differences ($p < 0.05$) determined by one-way analysis of variance (ANOVA) with Tukey's test.
